# Probing microwave fields and enabling in-situ experiments in a transmission electron microscope

**DOI:** 10.1038/s41598-017-11009-2

**Published:** 2017-09-11

**Authors:** F. J. T. Goncalves, G. W. Paterson, D. McGrouther, T. Drysdale, Y. Togawa, D. S. Schmool, R. L. Stamps

**Affiliations:** 1 Department of Physics and Electronics, Osaka Prefecture University, Osaka, 599-8570 Japan; 2 School of Physics and Astronomy, University of Glasgow, Glasgow, G12 8QQ UK; 3 Department of Engineering and Innovation, The Open University, Milton Keynes, MK7 6AA UK; 4 Groupe d’Etude de la Matière Condensée GEMaC, CNRS (UMR 8635), Université de Versailles/Saint-Quentin-en-Yvelines, 45 Avenue des États-Unis, 78035 Versailles, France

## Abstract

A technique is presented whereby the performance of a microwave device is evaluated by mapping local field distributions using Lorentz transmission electron microscopy (L-TEM). We demonstrate the method by measuring the polarisation state of the electromagnetic fields produced by a microstrip waveguide as a function of its gigahertz operating frequency. The forward and backward propagating electromagnetic fields produced by the waveguide, in a specimen-free experiment, exert Lorentz forces on the propagating electron beam. Importantly, in addition to the mapping of dynamic fields, this novel method allows detection of effects of microwave fields on specimens, such as observing ferromagnetic materials at resonance.

## Introduction

Knowledge of the electromagnetic field distribution of a given structure reveals information on its radiative and dielectric properties, which is important in areas spanning near-field communication technology and microwave assisted information processing and storage^1–3^. The mapping of electromagnetic fields produced by microwave circuits often represents great challenges in terms of spatial and temporal resolution. Over the past few decades, various forms of near-field microscopy enabled sub-wavelength mapping of microwave fields with ever improved spatial resolution^4–9^. However, typically they only allow field mapping of a given surface. The development of a probe for microwave fields in the environment of a TEM can be advantageous since electron microscopy allows a broad range of experiments, such as electric and magnetic contrast imaging, element sensitive or energy filtered spectroscopy with resolution down to the level of atoms. TEM has been employed to map the distribution of static electromagnetic fields in micro and nanometre sized structures by use of imaging methods such as differential phase contrast^10, 11^, Fresnel^12^, Focault^13^ and off-axis electron holography^1, 2, 14–17^. In wave-optics, the electron interaction with the field is described by the Aharonov-Bohm effect, whereby the electric and magnetic field distributions cause phase alterations to the transmitted electron waves which are converted into intensity contrast in the detector plane of the microscope by the various imaging methods mentioned^12, 16, 18–20^. When a TEM is operated in diffraction mode, the Fraunhofer diffraction condition is met by illuminating the sample with a parallel beam and thus the angle that the electron beam is scattered through can be recorded by imaging the back focal plane of the objective lens^21–23^. This plane is imaged by a detector coupled to a projection system, the magnification of which is determined by the equivalent ‘camera length’ through which the deflections are projected. The operating conditions of the microscope ultimately depend on the field strength to be detected. For Bragg diffraction due to the rapidly spatially varying (10^−10^ m) atomic lattice potential in crystalline materials, deflection angles are in the milliradian range and camera lengths of tens of centimeters are typically used. However, for more slowly spatially varying periodic objects (periodicity of 10^−8^–10^−9^ m) or fields such as the magnetic induction of thin ferromagnetic materials, the deflection angles can be as small as tens of microradians, requiring the use of larger camera lengths, typically in the order of 100–3000 metres. Under this condition, the technique is referred to as small angle electron scattering (SAES)^22, 23^ or low angle diffraction (LAD)^21^.

In this work, we investigate the electromagnetic (EM) field distribution of a loop shaped microstrip waveguide, operating in the gigahertz frequency range, using SAES. A special specimen holder was developed, capable of accommodating the microwave circuit and an impedance matched feed-through for the vacuum system. Microwave current flow in a loop shaped microstrip gives rise to a non-linear volume EM field distribution and the electron beam passing through this volume undergoes forces and torques associated with a space, time and frequency dependent EM fields. Due to the limited temporal resolution of the camera, the SAES patterns correspond to the time integrated polarisation state of the EM fields affecting the trajectory of the electron beam. Using the microwave EM field distribution from numerical simulations of the designed waveguide, we calculate the expected SAES patterns. These show the same main features seen in the experimental data, but with some small differences which we ascribe to imperfections in the realised circuit, highlighting the importance of being able to experimentally determine the field distribution.

## Results

**Figure 1:**
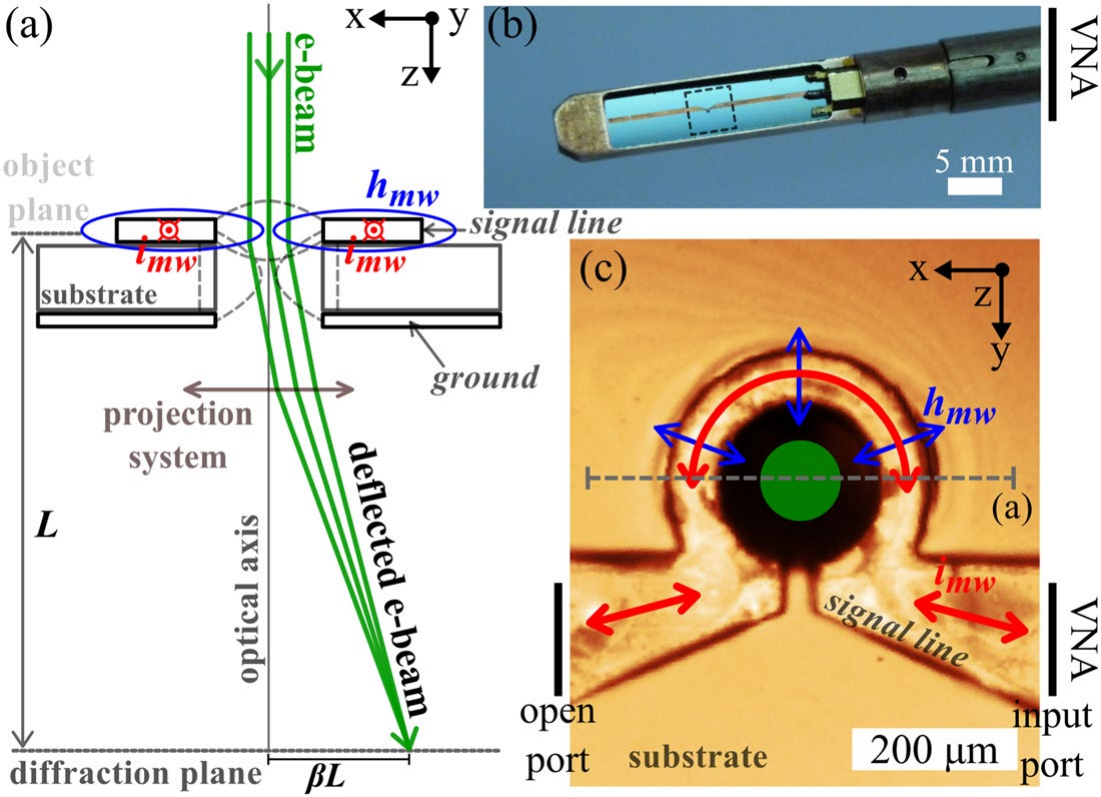
(**a**) Simplified schematic illustration of the imaging method. The electron beam travels through the region (object plane) with non-zero magnetic (illustrated by the blue lines) and electric fields (dashed gray lines), experiencing a net deflection. The non-zero magnetic and electric fields are a result of microwave (*i*_*mw*_) current flow in the microstrip signal line. The camera length (*L* = 410 m) corresponds to the magnified projection of the diffraction plane. (**b**) Optical micrograph showing the end-piece of the modified TEM specimen holder. The sample holder itself, constructed from phosphor bronze, was used as the ground for the waveguide. (**c**) Optical micrograph of the loop-shaped region of the microstrip. The width of the loop shaped signal line is 80 *μ*m, the hole diameter is 0.2 mm and the microstrip feed line is 0.5 mm wide. The red arrows illustrate the current flow in the signal line and the blue arrows illustrate the dominant microwave field components above the loop shaped signal line. The dielectric material enclosed by the signal line loop was etched to allow propagation of the electron beam. The green circle illustrates the area illuminated by the electron beam.

Figure 1(a) shows a simplified illustration of the SAES method. An electron beam travelling through a region (object plane) with non-zero electric and magnetic fields experiences a net deflection which is proportional to the local field strength integrated along the optical axis. The microwave current (*i*_*mw*_) flowing in the microstrip line gives rise to a spatially non-uniform electric and magnetic field distribution which exerts a Lorentz force on the electron beam. In SAES, as the TEM is operated in diffraction mode, regions of the area illuminated by the electron beam which experience the same net field strength will converge to the same point in the back focal plane of the objective lens. The diffraction plane is coupled to the image detector by a projection system and can be magnified by increasing the camera length, *L*.

The microstrip under study was designed to fit on the specimen holder of an FEI Tecnai 20 TEM equipped with a LaB_6_ thermionic electron gun, as illustrated in Fig. 1(b). Figure 1(c) shows details of the omega loop formed in the signal line and centred on the optical axis of the TEM column (see Fig. 1(a)). In order to allow the electron beam to propagate, a hole was etched through this section of the substrate. The loop shaped geometry was chosen so that one could obtain enhanced microwave fields in the area enclosed by the microstrip loop, as indicated by the field lines in Fig. 1(a) and (c), making it suitable for *in-situ* experiments. The overall length of the microstrip was chosen so that the circuit could be easily converted into a resonator by, for example, opening gaps in the signal line in positions equidistant from the loop centre, allowing the possibility of obtaining strong microwave fields at a specific frequency^24, 25^.

**Figure 2:**
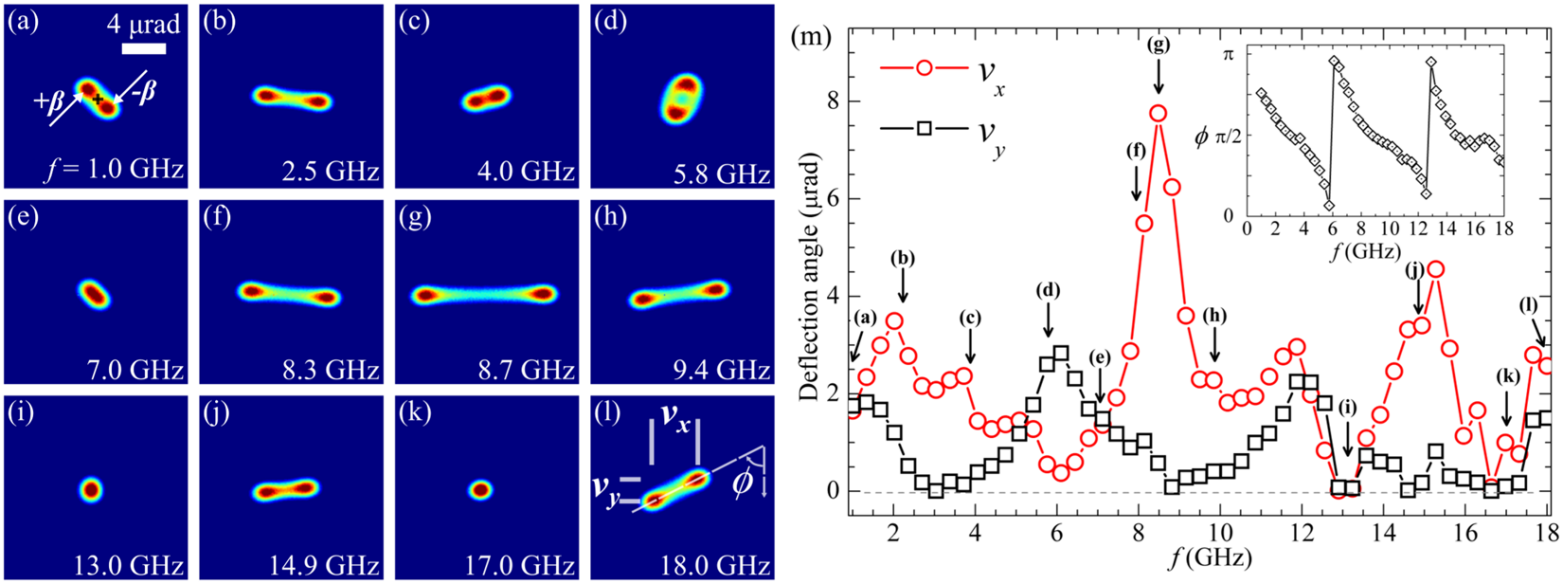
(**a**–**l**) SAES pattern of the electron beam transmitted through the circular hole in the waveguide at the indicated frequencies. In (**a**), the cross indicates the centre of the image, corresponding to the position of the optical axis. The quantity *β* indicates the net deflection angle. In (**l**), the terms *v*_*x*_ and *v*_*y*_ correspond to the deformation of the beam in the horizontal and vertical directions, respectively. The term *ϕ* represents the rotation angle of the SAES pattern long axis with respect to the vertical orientation. (**m**) Deflection angle, in microradians, of the SAES patterns projected along the *v*_*x*_ and *v*_*y*_ axes shown in (**l**), as function of frequency, *f*, of the microwave current. The inset shows the relative in-plane rotation angles, *ϕ*, of the SAES patterns.

In the experiments, the TEM was operated at 200 keV, the camera length was 410 m, and a vector network analyser (VNA), operated at 15 dBm, was used as a source and analyser of the microwave (MW) signal. A video of the SAES pattern was recorded while sweeping the VNA frequency from 1 to 18 GHz. The reflection coefficient of the circuit was monitored via the *S*_11_ scattering parameter measured by the VNA. Figure 2 shows a number of SAES patterns taken at different frequencies of the MW current applied to the circuit. The area illuminated by the electron beam is a fraction (~0.6) of the diameter of the inner loop (green circle in Fig. 1(c)). The SAES pattern consists of a diffraction spot deflected off the optical axis by an angle, *β*, proportional to the Lorentz force exerted on the electron beam. Importantly, because of the time dependence of the imaged fields, the diffraction spot will oscillate between +*β* and −*β* (Fig. 2(a)), while following a cos(*ω*t) dependence. As the frequency of the source increased, the SAES pattern continuously rotated and acquired different shapes, appearing elliptical at 5.8 GHz, elongated at 8.7 GHz, and at certain frequencies to be unaffected by the MW current, for example at 17.0 GHz. To quantify the shape and amplitudes of the beam deflections, we analysed the SAES patterns to extract components along the two orthogonal directions *v*_*x*_ and *v*_*y*_ indicated in Fig. 2(l). Figure 2(m) shows a summary of the extracted parameters as a function of frequency. The inset plot shows the rotation angle *ϕ* of the SAES pattern.

The shape of the SAES patterns directly reflects the net strength and polarisation state of the in-plane magnetic field and the electric field experienced by the electron beam as it propagates through the inner volume of the loop. A net effect can be a good approximation due to the very high speed of the electrons. The non-zero electric and magnetic fields affected the phase of the propagating electron beam in such a way that its SAES pattern was deflected off the optical axis, which is known as the Aharonov-Bohm effect^18^. It is instructive to compare the experimental results to those predicted by modelling. By using the electric and magnetic field distribution obtained from microwave simulations one is able to assess the accuracy of the model. Following ref. 14, the gradient of the electric potential and the magnetic induction field contribute to the phase of the electron wave and the resulting SAES pattern may be obtained by calculating the magnitude of the Fourier transform of the electron wave^12^ after being transmitted through the loop region.

**Figure 3:**
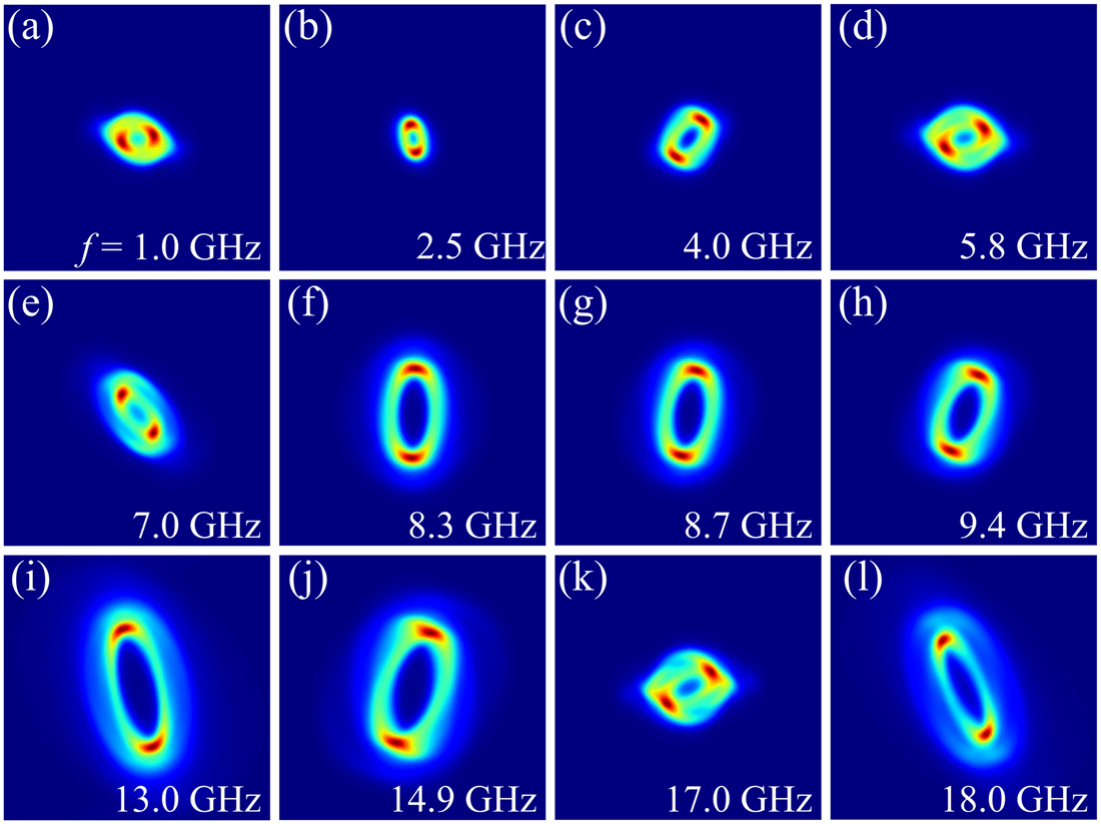
SAES patterns calculated using the electric and magnetic field distribution obtained from microwave simulations. A height dependent phase shift was considered as an approximation to the phase increment as the beam propagated through the region with non-zero EM fields. A top-hat mask was applied to the transmitted electron wave to mimic the diameter of the electron beam. The results shown are for uniform illumination and do not consider deformations in the image due to lens aberrations or the effective propagation distance of the beam across the TEM column.

Figure 3 shows the equivalent SAES patterns obtained from the simulation results, at the same frequencies as the experimental data. Numerical simulation results of the EM field distribution were obtained in the volume coincident with the electron trajectory and within a distance of 1 mm below and 8 mm above the plane of the signal line. Outside this range, the EM field amplitudes were a factor of 10^−3^ of those in the signal line plane. The SAES patterns were obtained by integrating the electron phase along the propagation direction and over one period of the oscillating MW current to replicate the time integrated data produced by the detector in the TEM experiments. The simulated data contains many of the same features seen in the experiment, including variation of the polarisation state, field amplitudes and rotation of the pattern with increasing frequency. The exact details, however, are not reproduced.

**Figure 4:**
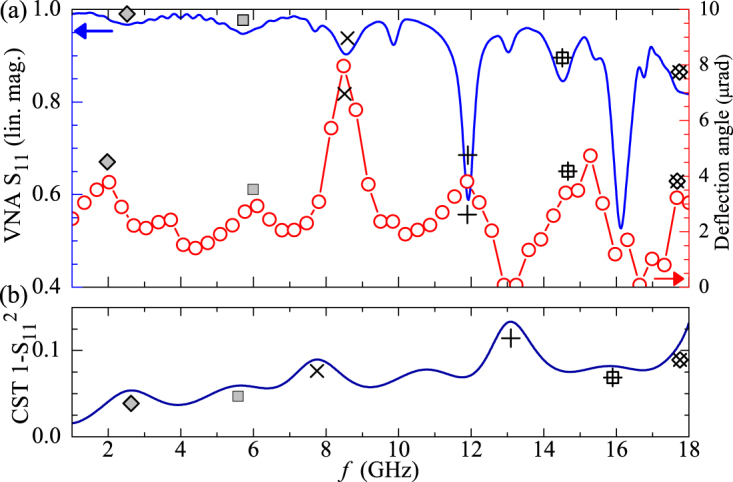
(**a**) Comparison between the magnitude of the electron beam deflection ($\sqrt{{v}_{x}^{2}+{v}_{y}^{2}}$) and the reflection coefficient, *S*_11_, measured with the VNA (smoothed). The short wavelength ripple observed in the *S*_11_ data can be caused by signal losses in the coaxial cables due to impedance mismatch. (**b**) Simulation results showing the power delivered to the microstrip (1-${S}_{11}^{2}$). The black symbols highlight similarities across the VNA, SAES and simulated CST spectra.

To understand the differences between the experimental and simulated data it is important to consider the impedance response of the microwave circuit, how it compares with the simulated impedance response, and how the differences between them affects the SAES patterns. The strength and polarisation of the local fields reflects the impedance condition imposed by loop shaped geometry and dielectric properties which define the local inductance and capacitance of the circuit. Figure 4 shows the electron beam deflection magnitude in the experiments and the reflection coefficient *S*_11_; which is a measure of the impedance measured in the VNA, and the simulated power delivered to the circuit. In ideal conditions, the power delivered to the circuit should correlate directly with the magnitude of the electron beam deflection described by the SAES data, and indeed there are similarities across the three data sets, indicated by black symbols. At frequencies below 10 GHz, the relative variation of the electron beam deflection follows both the attenuation of *S*_11_ and the trend in the simulated power delivered to the microstrip. However, at frequencies above 10 GHz, the agreement is less apparent as there is a pronounced increase in loss in the microwave circuit, indicated by the electron beam deflection becoming negligible at 13 and 16 GHz. This suggests that very little microwave power is present at the loop shaped region of the waveguide.

The discrepancy between the measured power dissipated by the circuit and the small deflection angle at certain frequencies can be explained by the presence of strong losses in other sections of the circuit, such as the vacuum feed-through, the surrounding metal parts and the coaxial cables. These imperfections are not taken into account in the ideal simulations where a constant power is delivered to the waveguide. This explains the difference between the simulated and experimental SAES patterns and highlights the importance of the ability to experimentally determine the field distribution.

From the viewpoint of circuit theory, the forward and backward waves propagating between the input port and the open port termination interfere, resulting in a standing wave with a defined amplitude and phase distribution. Part of this electromagnetic wave was returned to the VNA and measured via *S*_11_, while part was lost in the form of radiation or dielectric loss throughout the circuit. By using the electron beam as a probe we obtained information on the local field distribution which would otherwise be inaccessible by conventional network analyser measurements, as such methods only yield an overall response of the circuit. This method allows an understanding of the dynamic aspects of TEM imaging of micron and sub-micron features in the presence of electric and magnetic fields oscillating at gigahertz frequencies.

## Conclusions

In summary, we report the use of the TEM method of small angle electron scattering to probe the in-plane distribution of electric and magnetic fields at the gigahertz operating frequency of a loop-shaped microstrip waveguide operated in single port configuration. As the microwave frequency is varied, the SAES pattern acquired different intensity profiles as a result of the local amplitude and phase of the dynamic electric and magnetic fields, the detection of which is hardly accessible with other techniques. The frequency dependence of the SAES patterns correlated with the impedance of the circuit, measured with a VNA. However, the level of detail obtained on the local field amplitude and polarisation cannot be detected using VNA analysis, which highlights the importance of this TEM method.

Presently, our method does not allow time resolved imaging. In fact, only complex TEM adaptations enable time resolved imaging such as pump-probe TEM^26^. However, the prevalence of such instruments is low due to their complexity and the relative infancy of their development. One current drawback for this type of work is the vastly increased source size that results from the thermally assisted photoemission electron sources. In our view, *in-situ* microwave fields combined with the development of ever more efficient and highly time resolved imaging detectors^27^ will become an alternative as the small source size of continuous electron sources is maintained.

The method presented here can be used for high spatial resolution mapping of microwave fields as well as for studying the dynamic response of magnetic materials, thereby paving the way towards *in-situ* ferromagnetic resonance spectroscopy combined with the high spatial resolution inherent to the TEM. The simple extension of this technique to scanning-TEM has the potential to improve the spatial resolution needed to quantify properties of condensed matter.

## Methods

### Device fabrication

The microstrip waveguide was fabricated on a high impedance Si substrate (10 kΩ·m) using optical lithography followed by Si etching. The Si substrate was designed to conform to the specimen holder geometry and height limits within the confinement imposed by the TEM objective lens into which the holder must fit, and was 4.5 mm wide, 20 mm long and 0.525 mm thick. The VNA was connected to the waveguide via a series of coaxial cables and a vacuum feed-through. To enable TEM operation, a DC block was used to decouple the RF circuit ground from that of the TEM which is held at 2 V as part of a pole-touch detection mechanism.

The geometrical parameters of the microstrip waveguide were chosen to obtain an impedance of ~50 Ω which was verified using an EM simulation tool, CST Microwave Studio. The S_11_ calculation as well as the EM field distribution of the microstrip waveguide were determined using the frequency domain solver with a tetrahedral mesh (0.1 *μ*m resolution) and a convergence criteria of 0.02. Open boundary conditions on the outer limits of the waveguide structure were implemented. The substrate properties were set to lossless and without anisotropy. An effective dielectric constant of 8.65 was obtained within the frequency range examined.

## Electronic supplementary material

Supplementary information
