# Supplementary material for: Probing microwave fields and enabling in-situ experiments in a transmission electron microscope
Source: Sci Rep. 2017 Sep 11;7:11064. doi: 10.1038/s41598-017-11009-2 (PMC5593874; doi:10.1038/s41598-017-11009-2)
Supplement: Supplementary file 1 — Supplementary information [file 41598_2017_11009_MOESM1_ESM.pdf]

Probing microwave fields and enabling in-situ experiments in a transmission electron microscope

F. J. T. Goncalves[1,2,*], G. W. Paterson[2], D. McGrouther[2], T. Drysdale[3], Y. Togawa[1,2], D. S. Schmool[4], and R. L. Stamps[2]

[1]Department of Physics and Electronics, Osaka Prefecture University, Osaka 599-8570, Japan
[2]School of Physics and Astronomy, University of Glasgow, Glasgow G12 8QQ, UK
[3]Department of Engineering and Innovation, The Open University, Milton Keynes, MK7 6AA UK
[4]Groupe d'Etude de la Matière Condensée GEMaC, CNRS (UMR 8635), Université de Versailles/Saint-Quentin-en-Yvelines, 45 Avenue des États-Unis, 78035 Versailles, France
[*]f-goncalves@pe.osakafu-u.ac.jp

ABSTRACT

In Sec. I of the supplementary material we show the SAES pattern in the absence of current in the microwave circuit. In Sec. II we provide additional details on the theory of the Aharonov-Bohm effect which was used to calculate the SAES patterns from the simulated EM field data. Section III provides additional information about the nature of the oscillating EM fields in a microwave circuit. Two examples of the spatial distribution of the calculated in-plane EM fields at two different frequencies are shown. The dynamic aspect of the SAES patterns is demonstrated in an example showing the time (phase) evolution of an SAES pattern as result of the calculated EM field distribution at a given frequency. Finally, Sec. IV provides some insight on future experiments to be carried out using this novel technique.

A technique is presented whereby the performance of a microwave device is evaluated by mapping local field distributions using Lorentz transmission electron microscopy (L-TEM). We demonstrate the method by measuring the polarisation state of the electromagnetic fields produced by a microstrip waveguide as a function of its gigahertz operating frequency. The forward and backward propagating electromagnetic fields produced by the waveguide, in a specimen-free experiment, exert Lorentz forces on the propagating electron beam. Importantly, in addition to the mapping of dynamic fields, this novel method allows detection of effects of microwave fields on specimens, such as observing ferromagnetic materials at resonance.

I: SAES pattern of undeflected electron beam, in the absence of microwave signal

Figure 2(a) shows an SAES pattern taken while the microwave source is switched off (P_{OFF}) (no microwave current flowing in the circuit). In the absence of microwave fields, the SAES pattern consists of a circular spot centred in the diffraction plane. For immediate comparison Fig.2(b) shows an SAES pattern acquired while the microwave power (P_{ON}) is set to 15 dBm at an operating frequency of 1 GHz.

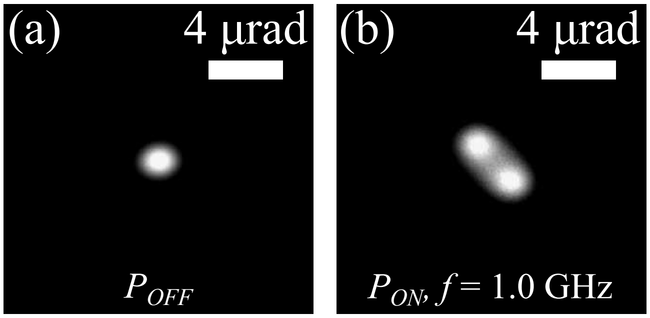

Figure 1. SAES pattern of the electron beam while the microwave power source is (a) switched off and (b) set to 15 dBm at an operating frequency of 1 GHz.

II: Theoretical considerations

At the core of the electron beam interaction with EM fields lies the Aharonov-Bohm effect. A beam of coherent electrons propagating along the optical axis z of an electron microscope can be interpreted as a monochromatic plane wave $\Psi = Ae^{ik_z \cdot \vec{z}}$ with complex amplitude $A = A_0(r,z)e^{i\phi(r,z)}$. The effects of propagating the electron beam in a region with non-zero electric and magnetic potentials can be expressed as a phase gradient $\nabla\vec{\phi}(r,z)$, which is given by[1–5]:

$$\nabla\phi(x,y) = C_U \nabla(t\vec{V}(r)) - \frac{e}{\hbar}[\hat{n}_z \times t\vec{B}(r)] \tag{1}$$

where the constant C_U is given by $C_U = \left(\frac{2\pi}{\lambda}\right)\left(\frac{U+U_0}{U(U+2U_0)}\right)$. The value of C_U is 7.28×10^6 rad/Vm at 200 keV electron beam[1,6]. The term λ corresponds to the electron wavelength. The terms U and U_0 correspond to the kinetic energy and rest mass energy of the electrons, respectively. The term t corresponds to the distance over which the EM field is non-zero and $\hat{n}_z$ the unit vector parallel to the propagation direction of the electrons. The first term on the right accounts for the gradient of the electric potential $\vec{V}(r)$ ($\vec{E} = -\nabla\vec{V}$) and the second term accounts for the induction field components perpendicular to propagation direction.

The intensity distribution of an image obtained with SAES corresponds to a magnified view of the pattern formed in the back focal plane of the image forming lens[3]. In this plane, the electron wave is given by the Fourier transform $\mathscr{F}[A_0 e^{i\nabla\phi \cdot \vec{z}}]$ of the wave transmitted through the region with non-zero EM fields. Thus the intensity pattern imaged in SAES corresponds to the magnitude of $|\mathscr{F} A_0 e^{i\nabla\phi \cdot \vec{z}}|^2$.

Figure 1 illustrates the formation of a diffraction pattern resulting from an 'ideal' specimen with two magnetic domains, whose magnetic moment is in opposite direction, and separated by a domain wall (neglected). The SAES pattern was obtained following the implications of Eq. 1.

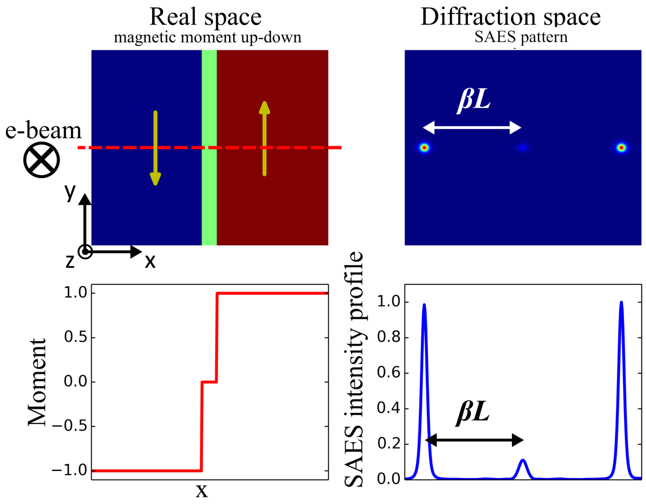

Figure 2. Illustration of the effect of two magnetic domains on the electron beam. Two magnetic domains opposed to each other (left) induce a phase variation on the transmitted electron beam. When the image is acquired in the back focal plane of the image forming lens, the pattern observed resembles the image shown on the right, which is known as an SAES pattern. From a classical perspective, the electron beam experiences a Lorentz deflection angle β proportional to the layer thickness and the magnetic induction, as expressed in Eq. 2. The separation between the two spots on the image corresponds to $2\beta L$, with L being the camera length. The low intensity signal at the centre of the SAES pattern corresponds to the undeflected beam which crossed the domain wall where the net induction field is assumed negligible.

In cases where the magnetic induction can be considered uniform across the film thickness and the gradient of the electric potential may be neglected, the diffraction pattern can be described as two intensity spots deflected by an angle β. The deflection angle, β, is directly proportional to the thickness, t, of the region with non-zero magnetic induction, B_0, as expressed in Eq. 2.

$$\beta = \frac{e\lambda B_0 t}{h} \tag{2}$$

When both the gradient of the electric potential and the magnetic induction field are considered, complex SAES patterns emerge, which is the case of the present study. When calculating the simulated SAES patterns one required the approach given by Eq. 1, as explained in the following section.

III: Simulated SAES patterns

Having discussed an example of the static field distribution, it is now relevant to discuss the nature of dynamic EM fields in the microwave frequency range. EM fields in the microwave frequency range are time and space varying waves whose electric (and magnetic) field component can be expressed as[7]:

$$\vec{E} = \Re\{\vec{E}_0(x,y,z)e^{i\omega t - \vec{k}.\vec{r}}\}, \tag{3}$$

where E_0 is the wave amplitude, ω is the angular frequency and k represents the propagation vector. For simplicity, assume that $\vec{E}$ and $\vec{H}$ are transverse wave modes so the field components are along $\hat{x}$ and $\hat{y}$. If only the time varying components of Eq. 3 are considered (fixed z= 0), the electric field can be written as

$$\vec{E} = \hat{x}E_0^x\cos(\omega t) + \hat{y}E_0^y\sin(\omega t). \tag{4}$$

Note that at a fixed position, take $z = 0$, the electric field direction rotates with ωt following its orthogonal field components in what is defined as a polarisation state. The relative amplitude of the E_0^x and E_0^y as well as the phase, given by

$$\phi = tan^{-1}\frac{E_0^y\sin(\omega t)}{E_0^x\cos(\omega t),} \tag{5}$$

will define the polarisation of the electric field. EM waves can be described as linearly polarized if either E_0^x or E_0^y components are zero or $\phi = \pi/4$ radians. The waves will have circular polarisation if $E_0^x = E_0^y$ and $\phi = \pi/2$ radians. Elliptical polarisation will be obtained for $E_0^x \neq E_0^y$.

In a microstrip with non-linear shape, such as loops or bends, the polarisation state will not be a linear polarisation since propagating waves cannot be approximated to transverse EM modes and their propagation direction cannot be trivially determined since the propagating modes in the loop shaped region of the microstrip are hybrid transverse electric (TE) and transverse magnetic TM) modes. As a consequence, when probing the EM field distribution in the SAES experiments one should expect a superposition of polarisation states. Figures 3(a) and 3(b) show examples of the calculated spatial distribution of the in-plane electric and magnetic field components as well as the corresponding field magnitude at 1 GHz and 8.7 GHz, respectively, in the region imaged by the electron beam.

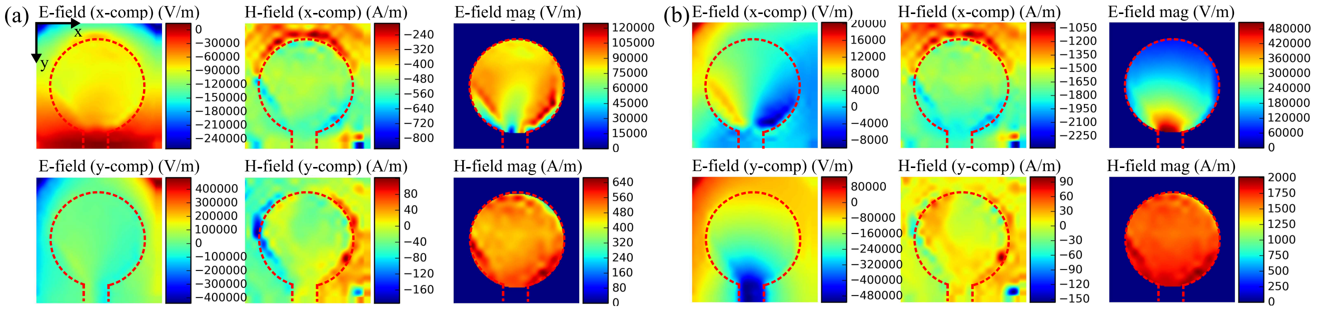

Figure 3. Examples of the spatial distribution of the electric and magnetic field in the directions perpendicular to the electron beam path at f of 1 and 8.7 GHz. The dashed lines indicate the inner edge of the looped microstrip line. The phase term ωt is set to zero.

Having obtained the distribution of the electric and magnetic field from the microwave simulations we calculated the corresponding SAES patterns by applying Eq. 1 and integrating the phase along the propagation direction of the electron beam. The final SAES patterns were obtained by integration over a 2π rotation of the phase term corresponding to ωt. Figure 4 shows the evolution of the SAES pattern as the phase varies from 0 to 2π in phase steps of 0.3 radians at 18 GHz. The calculated SAES pattern shown in Fig. 4(u) corresponds to the time-integrated intensity profile, resembling the SAES profiles obtained in the experiments.

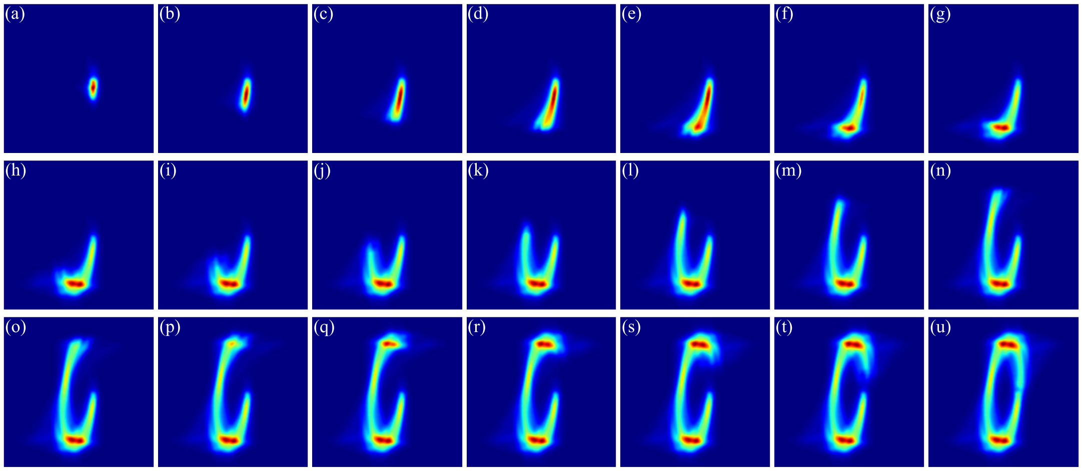

Figure 4. Intensity profile of the simulated SAES pattern as the phase of the EM fields vary from 0 to 2π in phase steps of 0.3 radians. The SAES pattern shown in (u) corresponds to the time-integrated intensity measured the experiments.

IV: Future perspectives

The simple extension of this technique to scanning-TEM has the potential to improve the spatial resolution needed to quantify properties of condensed matter which are hardly accessible. For example, one can (1) characterise the ground state of the specimen while driving its local or non-local precessional modes; (2) measure deformations in the SAES patterns while the specimen in at resonance and correlate these with the lineshape of the resonance response and (3) study microwave field switching on nano-structured magnetic materials. From a different view point, the control over the polarisation state would enable the study of materials whose slow and fast response is very sensitive to the driving microwave field polarisation[8].

References

1. Volkov, V. V. & Zhu, Y. Phase Imaging and Nanoscale Currents in Phase Objects Imaged with Fast Electrons. *Phys. Rev. Lett.* **91**, 043904 (2003). URL https://link.aps.org/doi/10.1103/PhysRevLett.91.043904. DOI 10.1103/PhysRevLett.91.043904.
2. Aharonov, Y. & Bohm, D. Further Considerations on Electromagnetic Potentials in the Quantum Theory. *Phys. Rev.* **123**, 1511–1524 (1961). URL https://link.aps.org/doi/10.1103/PhysRev.123.1511. DOI 10.1103/PhysRev.123.1511.
3. Chapman, J. N. The investigation of magnetic domain structures in thin foils by electron microscopy. *J. Phys. D: Appl. Phys.* **17**, 623–647 (1984). URL http://stacks.iop.org/0022-3727/17/i=4/a=003?key=crossref.5aae3c90eeb5783e90dc3e40a8f77c80. DOI 10.1088/0022-3727/17/4/003.
4. Shindo, D. & Murakami, Y. Electron holography study of electric field variations. *Microsc.* **60**, S225–S237 (2011). URL https://academic.oup.com/jmicro/article-lookup/doi/10.1093/jmicro/dfr017. DOI 10.1093/jmicro/dfr017.
5. Eskin, G. Aharonov–Bohm effect revisited. *Rev. Math. Phys.* **27**, 1530001 (2015). URL http://arxiv.org/abs/1504.04784. DOI 10.1142/S0129055X15300010. 1504.04784.
6. Volkov, V. V. & Zhu, Y. Lorentz phase microscopy of magnetic materials. *Ultramicroscopy* **98**, 271–281 (2004). URL http://linkinghub.elsevier.com/retrieve/pii/S0304399103002079. DOI 10.1016/j.ultramic.2003.08.026.
7. Pozar, D. *Microwave Engineering* (2005). URL http://scholar.google.com/scholar?hl=en&btnG=Search&q=intitle:No+Title#0.
8. Goncalves, F. J. T. *et al.* Collective resonant dynamics of the chiral spin soliton lattice in a monoaxial chiral magnetic crystal. *Phys. Rev. B* **95**, 104415 (2017). URL https://link.aps.org/doi/10.1103/PhysRevB.95.104415. DOI 10.1103/PhysRevB.95.104415.
